# Innovative antifungal strategies to combat drug-resistant *Candida auris*: recent advances and clinical implications

**DOI:** 10.3389/fcimb.2025.1641373

**Published:** 2025-07-31

**Authors:** Wei Du, Qihui Wang, Min Zhao

**Affiliations:** ^1^ National Clinical Research Center for Laboratory Medicine, Department of Laboratory Medicine, The First Hospital of China Medical University, Shenyang, China; ^2^ Department of Laboratory Medicine, The First Hospital of China Medical University, Shenyang, China

**Keywords:** antifungal resistance, *Candida auris*, alternative antifungal therapies, natural compounds, nanotechnology delivery, combination therapies, photodynamic therapy

## Abstract

*Candida auris* is an invasive fungal pathogen recognized globally as a significant health threat due to its marked resistance to multiple classes of antifungal agents, including azoles, echinocandins, and polyenes. The associated high morbidity and mortality rates present considerable public health challenges. Research efforts have largely focused on understanding the molecular mechanisms of antifungal resistance and developing alternative therapies to counteract this issue. This review summarizes current advances in the identification of natural antifungal compounds, the development of novel synthetic agents, biological antifungals, nanotechnology-based approaches, combination therapies, and photodynamic treatments. Notably, several synthetic compounds such as rezafungin and fosmanogepix are in clinical trials for *C. auris* infections. Biological antifungals, including monoclonal antibodies, vaccines, and peptides, have shown the capacity to enhance host immune responses and reduce mortality in murine models. Combination therapies have proven particularly valuable for overcoming resistance by exploiting synergistic effects and broadening antimicrobial coverage. Despite these promising developments, majority of studies have been conducted *in vitro*, with a relative lack of *in vivo* or human research. Therefore, further investigation is needed to validate the efficacy and safety of these alternative antifungal strategies for the treatment of drug-resistant *C. auris* infections.

## 1 Introduction

*Candida* species remain a leading cause of opportunistic fungal infections, with mortality rates exceeding 40% in invasive candidiasis (Pfaller and Diekema, 2007; Chen et al., 2021). A comprehensive global review estimates that between 250,000 and 700,000 cases of candidemia and invasive candidiasis occur annually worldwide. The rates of candidemia in hospital settings demonstrate a consistent prevalence, with incidence rates in recent years ranging from approximately 0.022% to 0.029% of hospitalized patients (Dai et al., 2025; Mallick et al., 2025). The species of *Candida* most frequently identified is *C. albicans*; however, non-albicans species are increasingly observed. *Candida* infections are prevalent among critically ill and hospitalized patients, significantly contributing to global morbidity and mortality (Cornely et al., 2025; Khan et al., 2025; Mallick et al., 2025). *Candida auris* is a highly concerning fungus within this genus due to its notable antifungal resistance. This organism was first isolated from the ear canal of a hospitalized patient in Japan in 2009, and since that time, it has disseminated globally (Satoh et al., 2009; Eix and Nett, 2025). Under normal circumstances, *C. auris* is part of the human skin flora without causing infection. However, *C. auris* can induce bloodstream infections that may lead to invasive diseases, particularly due to the presence of medical devices and catheters, a compromised immune system, and prolonged stays in an intensive care unit (Adams et al., 2018; Park et al., 2019; Shastri et al., 2020). According to data from the Centers for Disease Control and Prevention (CDC), there were over 2,377 reported clinical cases of *C. auris* in the United States, with new clinical cases increasing to 4,515 in 2023 (Hayes, 2024; Bhargava et al., 2025; Mallick et al., 2025). In a five-year continuous study conducted from 2017 to 2022 in United States hospitals, the mortality rate associated with 192 cases of *C. auris* infection was as high as 34%. In comparison, reported mortality rates in hospitals in Europe, Pakistan, and India were 41.4%, 62.6%, and 19.6%, respectively (Chakrabarti et al., 2015; Ruiz-Gaitan et al., 2018; Sayeed et al., 2020). Moreover, in hospital mortality rates associated with *C. auris* bloodstream infections have been reported to range from 30% to 72% across various studies (Cortegiani et al., 2018; Chowdhary et al., 2023). In addition to the significantly high mortality rate associated with *C. auris* infections, another critical factor of concern is the organism’s drug resistance. According to data from the CDC, the tentative minimum inhibitory concentration (MIC) breakpoints (in μg/ml) have been established as follows: fluconazole ≥ 32, amphotericin B (AmB) ≥ 2, anidulafungin ≥ 4, caspofungin ≥ 2, and micafungin ≥ 4 (CDC, 2024). According to the antifungal susceptibility testing data obtained from the SENTRY Antifungal Surveillance Program, which collected 78 C*. auris* isolates from various anatomical sites of patients across different countries, it was found that 82.1% of the strains exhibited resistance to fluconazole, 17.9% demonstrated resistance to AmB, and 1.3% was resistant to caspofungin. This extensive study indicates that fluconazole resistance is highly prevalent on a global scale, while resistance to other antifungal agents, such as AmB and echinocandins, is also observed, albeit at a lower frequency (Castanheira et al., 2024). Clinical strains that exhibit resistance to three or even four classes of antifungal agents are referred to as pan-resistant *C. auris* (Ostrowsky et al., 2020; Jacobs et al., 2022). The CDC has classified *C. auris* as an urgent threat due to its rapid dissemination and frequent resistance to antifungal treatments. While resistance to echinocandins remains relatively uncommon, it is on the rise and poses significant concern. The emergence of resistance to multiple antifungal agents complicates treatment strategies and raises substantial public health issues (CDC, 2025). The recent emergence of pan-resistant *C. auris* strains has highlighted significant deficiencies in conventional antifungal therapies that target ergosterol biosynthesis (azoles), cell membrane integrity (polyenes), and β-glucan synthesis (echinocandins) (Forsberg et al., 2019; Lima et al., 2019; Vila et al., 2020; Vitiello et al., 2023). This therapeutic crisis has prompted the exploration of alternative strategies, including natural phenolic compounds, engineered nanoparticles, and monoclonal antibodies. Briefly, over 90% of *C. albicans* biofilm was inhibited by the combination of a curcumin derivative (a natural phenolic compound) and fluconazole. Most antifungal drugs have poor solubility, and engineered nanoparticles (NPs) feature a hydrophilic outer membrane and a hydrophobic core to encapsulate these poorly soluble drugs (Wu et al., 2025). Additionally, engineered NPs reduce cytotoxicity and enhance the antifungal efficacy of antifungal drugs. Maciel-Magalhaes et al. demonstrated that encapsulating amphotericin B (AmB) in polycaprolactone (PCL) and polylactic acid (PLA) polymeric NPs results in lower adverse effects compared to the free (unencapsulated) drug. This encapsulation protects non-target tissues from exposure to high concentrations of free AmB, thereby decreasing cytotoxicity and renal side effects while maintaining antifungal efficacy (Maciel-Magalhaes et al., 2025). Furthermore, research conducted by Seth et al. in 2024 highlighted that an engineered nanoformulation of AmB improved drug targetability and bioavailability, leading to increased antifungal efficacy with significantly reduced toxicity compared to traditional formulations (Seth et al., 2024). Additionally, combination therapies that leverage Food and Drug Administration (FDA)-approved antifungals alongside novel adjuvants show promise in overcoming resistance mechanisms through synergistic action. This review systematically evaluates these emerging strategies in terms of mechanistic innovation, preclinical efficacy, and clinical feasibility.

## 2 Novel antifungal drugs and strategies

### 2.1 Natural compounds as antifungal agents

Natural products have long been a rich source of antifungal agents. Recent studies have identified novel compounds, such as terpenoids and alkaloids, with potent antifungal activity (Zacchino et al., 2017; Ganeshkumar et al., 2023; Honorato et al., 2024) (**Table 1**). A recent study demonstrated that a derivative of the natural compound nikkomycin Z exhibited strong inhibitory effects against *C. auris* and other resistant strains (Bentz et al., 2021). Although the inhibition of this competitive chitin synthase inhibitor (which hinders fungal cell wall synthesis) has only been tested *in vitro*, it offers a novel antifungal agent against *Candida* species (Adnan et al., 2023). Berberine is an alkaloid extracted from *Berberis vulgaris* that exhibits broad-spectrum antifungal activities through mechanisms such as membrane disruption, reactive oxygen species (ROS) generation, biofilm inhibition, and mitochondrial dysfunction (Li and Calderone, 2017; Ding et al., 2024). Recent studies have demonstrated the efficacy of this natural compound in targeting drug-resistant *Candida* species, with berberine showing significant potential in overcoming multidrug resistance in *C. albicans* by inhibiting the expression of the efflux pump *MDR1* gene (Tong et al., 2021). Additionally, berberine displays remarkable antifungal efficacy against fluconazole-resistant *C. albicans* by upregulating the expression of the *ATP11* gene and downregulating *SOD2*, which leads to increased ROS generation (Huang et al., 2022). By downregulating the expression of efflux pump genes *CDR1* and *MDR*, as well as hypha growth-related genes, berberine significantly enhances the antifungal effects of fluconazole when used synergistically with this antifungal agent (Wu et al., 2024). Although berberine has been studied for its antifungal efficacy against various *Candida* species, such as *C. albicans* and *C. glabrata*, its effectiveness against *C. auris* has not been fully established (Xie et al., 2020; Gupta et al., 2023). This antifungal agent may hold great promise for treating multidrug-resistant strains of *C. auris* (Liu et al., 2020). Another significant family of natural compounds that demonstrate antifungal effects against drug-resistant strains is phenolics, which includes curcumin (found in turmeric), eugenol (derived from clove oil), and resveratrol (present in grapes) (Moghadamtousi et al., 2014; Marchese et al., 2017; Vestergaard and Ingmer, 2019). Interestingly, these three natural compounds exhibit distinct actions against fungi. Curcumin inhibits fungal biofilm formation, eugenol disrupts cell wall synthesis, and resveratrol interferes with inhibiting of yeast-hyphae morphological transition (Okamoto-Shibayama et al., 2010; Kerekes et al., 2013; Houille et al., 2014; Marchese et al., 2017; Dong et al., 2021; Lima et al., 2025). Although the antibacterial and antiviral properties of resveratrol have been established, its antifungal effects require further elucidation, as several studies have yielded conflicting results (Collado-Gonzalez et al., 2012). Furthermore, the molecular mechanisms underlying its antifungal activity remain inadequately investigated. Additionally, Jin’s laboratory identified a derivative of curcumin (Compound 4) that exhibits significant inhibition of biofilm formation and the yeast-to-hypha morphological transition. Furthermore, the synergistic combination of fluconazole and Compound 4 demonstrated over 90% anti-biofilm efficacy (32 μg/ml of Compound 4 combined with 4 μg/ml of fluconazole). Additionally, the use of these two antifungal agents inhibited nearly 90% of ATP production (Dong et al., 2021). Although this study focuses on fluconazole-resistant *C. albicans*, it offers a novel approach for utilizing curcumin to inhibit biofilm formation in *C. auris.* These findings underscore the diverse mechanisms and therapeutic potential of natural products—including terpenoids, alkaloids, phenolics, and chitin synthase inhibitors—as promising antifungal candidates against drug-resistant fungal pathogens.

**Table 1:** Antifungal strategies utilizing natural compounds.

Natrual compounds	Antifungal mechanisms	Outcomes	References
Alkaloids (e.g. solenopsin)	Inhibit matrix deposition; Disrupt biofilm formation; Compromise membrane integrity;	Against six CDC strains of *C. auris in vitro*, which are resistant to Flu and AmB, with an IC_50_ range of 0.7 to 1.4 µg/ml. The combination of solenopsins with AmB enhances the susceptibility of AmB-resistant strains of *C. auris* Solenopsins improve the survival rate of *Galleria mellonella* larvae infected with *C. auris.*	(Honorato et al., 2024)
Alkaloids (e.g. Berberine)	Disrupt fungal membrane; Against Flu-resistant *C. albicans* strains by upregulating the expression of *ATP11* and downregulating *SOD2* to increase ROS generation; Deregulating the expression of hypha growth-related genes; Inhibit biofilm formation; Reduce expression of drug resistance related genes *C. albicans* strains: *MDR1*and *CDR1*	Decreased antifungal drug resistance Enhanced the fungal growth inhibition when used synergistically with Flu	(Li and Calderone, 2017; Tong et al., 2021; Ding et al., 2024; Wu et al., 2024)
Nikkomycin Z	Competitive inhibit the chitin synthase	Exhibit antifungal effects to over 100 Flu, echinocandins and AmB resistant strains of *C. auris*	(Bentz et al., 2021; Adnan et al., 2023)
Phenolics (curcumin)	Inhibit fungal biofilm formation Inhibit of yeast-hypha morphological transition Disrupt ATP production when combine with Flu	The synergistic combination of Flu and curcumin demonstrated over 90% anti-biofilm efficacy (32 μg/ml of curcumin combined with 4 μg/ml of Flu).	(Moghadamtousi et al., 2014; Dong et al., 2021)
Phenolics (eugenol)	Disrupt cell wall synthesis Disrupt cell membrane fluidity and permeability Inhibit of yeast-hypha morphological transition Inhibit H^+^-ATPase activity	The mean MIC values of eugenol were found to be 625 μg/ml against *C. albicans* and 293 μg/ml against *C. neoformans*, respectively Inhibit the growth of *Aspergillus terreus* at 140 μg/ml Inhibit 90% of *C. glabrata* growth occurs at concentration levels approximately 1.5 times lower than Flu required to achieve a half inhibition of fungal growth.	(Marchese et al., 2017)
Phenolics (resveratrol)	Inhibit of yeast-hypha morphological transition	The MIC for *C. albicans* is range from 10 to 37 µg/ml	(Jung et al., 2005; Okamoto-Shibayama et al., 2010; Houille et al., 2014; Vestergaard and Ingmer, 2019)

Flu, fluconazole; AmB, Amphotericin B; *MDR1*, Multidrug resistance 1; *CDR1*, Candida Drug Resistance 1; *SOD2*, Superoxide dismutase 2; MIC, minimum inhibitory concentrations; IC_50_, Half-maximal inhibitory concentration.

### 2.2 Novel synthetic antifungal compounds

Synthetic antifungal compounds, such as azoles, polyenes, and echinocandins, are widely used to combat fungal infections. However, the prolonged administration and misuse of these drugs can lead to significant drug resistance and side effects, resulting in severe consequences. Therefore, the primary objective of the next generation of antifungal drugs is to maintain the efficacy of fungal eradication while minimizing the development of resistance as much as possible (**Table 2**). For example, the newer triazole, isavuconazole, is currently being investigated for its potential to overcome resistance mechanisms in *C. auris* (Nagy et al., 2021). Isavuconazole demonstrates the ability to overcome drug resistance in *C. auris* through mechanisms that are distinct from those of conventional azoles, such as fluconazole. Specifically, the unique molecular structure of isavuconazole contributes to its broader spectrum of binding affinity for lanosterol 14α-demethylase and enhances its stability, resulting in reduced susceptibility to mutations in the *ERG11* gene and the overexpression of efflux pumps. These characteristics enable isavuconazole to effectively address both intrinsic and acquired resistance mechanisms in *C. auris*, positioning it as a promising alternative in cases where conventional antifungal agents exhibit diminished efficacy (Logan et al., 2022; Jangir et al., 2023). Moreover, there are several antifungal drugs are under investigation to overcome conventional drug resistance, including rezafungin, ibrexafungerp, fosmanogepix, olorofim, and opelconazole (Hoenigl et al., 2021; Espinel-Ingroff and Wiederhold, 2024). Rezafungin is classified to the member of echinocandins and under two phase III trials (ReSTORE, NCT03667690 and ReSPECT, NCT04368559). Rezafungin inhibits the activity of β-1, 3-D-glucan synthase, which disrupts the synthesis of the fungal cell wall (Garcia-Effron, 2020). In comparison to conventional echinocandins, such as caspofungin, rezafungin exhibits enhanced pharmacodynamics and pharmacokinetics, along with reduced hepatotoxicity (Ong et al., 2016). In *in vitro* tests, the results of the minimum inhibitory concentrations (MIC) demonstrated that rezafungin exhibited better activity against drug-resistant strains of *C. auris* compared to other echinocandins (Helleberg et al., 2020). Ibrexafungerp is a novel echinocandin that acts as a glucan synthase inhibitor and can be administered orally. This antifungal drug exhibits significant fungicidal activity against *Candida* species, including *C. auris*. Because the binding sites of ibrexafungerp on β-1, 3-D-glucan synthase are distinct from those of caspofungin, this antifungal agent shows great potential to circumvent cross-resistance with conventional echinocandins (Walker et al., 2011; Jimenez-Ortigosa et al., 2017). Fosmanogepix is another novel synthetic antifungal agent that is converted into the active form, manogepix, once administered to humans (Shaw and Ibrahim, 2020). This antifungal agent aims to inhibit the Gwt1 enzyme, which hinders the transport of mannoproteins to the fungal cell wall and membrane, thereby causing a loss of stability in fungal cells (Miyazaki et al., 2011). Clinically, this antifungal agent exhibits great potential for treating *C. auris* candidemia, and the survival rate of infected patients has significantly improved (Hoenigl et al., 2021). A multicenter, open-label, single-arm Phase 2 clinical trial was conducted in the United States in 2023. A total of 20 patients were recruited, and the survival rate reached 85% following 30 days of treatment, with no reported deaths related to fosmanogepix. Furthermore, no severe adverse effects were observed, indicating that this antifungal agent demonstrates promising safety and efficacy in the treatment of candidemia caused by the multidrug-resistant pathogen *C. auris* (Vazquez et al., 2023). The other two novel antifungal drugs are olorofim and opelconazole, which act as inhibitors of dihydroorotate dehydrogenase and lanosterol 14α-demethylase. These enzymes are crucial for maintaining the integrity of the fungal cell membrane; their inhibition ultimately leads to the suppression of fungal cell growth. Olorofim and opelconazole are newly developed antifungal drugs that belong to the dihydroorotate dehydrogenase enzyme inhibitor and triazole families, respectively. They have been optimized for oral and inhalation administration to treat infections caused by *Aspergillus* species (Kriegl et al., 2024). Additionally, several studies have demonstrated that olorofim possesses a strong ability to treat azole-resistant strains of *Aspergillus fumigatus* by inhibiting fungal cell growth and biofilm formation (Buil et al., 2022; Kirchhoff et al., 2022; van Rhijn et al., 2022). Thus, the research findings may provide novel strategies and perspectives for treating drug-resistant strains of *C. auris*. In conclusion, the development of next-generation antifungal agents, such as isavuconazole, rezafungin, and ibrexafungerp represents a critical strategy for balancing therapeutic effectiveness with the reduction of resistance development in challenging pathogens such as *C. auris* and *A. fumigatus*.

**Table 2:** Antifungal strategies utilizing novel synthetic antifungal compounds.

Novel antifungal compounds	Novel antifungal effects	Outcomes	References
Isavuconazole	Different structures from conventional azoles that with enhanced binding affinity and stability for lanosterol 14α-demethylase.	Reduced susceptibility to mutations in the *ERG11* gene and the overexpression of efflux pumps; Overcome intrinsic and acquired resistance mechanisms in *C. auris*	(Nagy et al., 2021; Logan et al., 2022; Jangir et al., 2023)
Rezafungin	Inhibits the activity of β-1, 3-D-glucan synthase, which disrupts the synthesis of the fungal cell wall; Enhanced pharmacodynamics and pharmacokinetics, along with reduced hepatotoxicity	Exhibit better activity against drug-resistant strains of *C. auris* compared to other echinocandins	(Ong et al., 2016; Garcia-Effron, 2020; Helleberg et al., 2020)
Ibrexafungerp	Alternative binding site to β-1, 3-D-glucan synthase from other caspofungin Orally administered	Exhibit significant fungicidal activity against *Candida* species, including *C. auris*. Exhibit great potential to circumvent cross-resistance with conventional echinocandins	(Walker et al., 2011; Jimenez-Ortigosa et al., 2017)
Fosmanogepix	Disrupt the transport of mannoproteins to the fungal cell wall and membrane via the inhibition of Gwt1 enzyme; No severe adverse effects were observed	Exhibit great potential for treating *C. auris* candidemia, and the survival rate of infected patients has significantly improved The survival rate among the 20 patients was 85% after 30 days of treatment, with no deaths reported that were associated with fosmanogepix.	(Miyazaki et al., 2011; Shaw and Ibrahim, 2020; Hoenigl et al., 2021; Vazquez et al., 2023)
Olorofim	Inhibit activities of dihydroorotate dehydrogenase and lanosterol 14α-demethylase. Disrupt cell wall integrity of the fungal cell membrane	Inhibit fungal cell growth and biofilm formation in azole-resistant strains of *Aspergillus fumigatus*	(Buil et al., 2022; Kirchhoff et al., 2022; van Rhijn et al., 2022)
Opelconazole		Designed for inhalation and exhibits promise as an effective agent for the treatment of pulmonary aspergillosis It inhibits over 96 clinically isolated strains of A. fumigatus and exhibits 2.5-fold greater potency compared to Voc, while demonstrating comparable potency to Pos. Exhibit broad-spectrum antifungal activity against *C. albicans, C. auris, C. krusei, C. glabrata, C. neoformans*, and *Aspergillus* spp.	(Colley et al., 2017; Cass et al., 2021; Hoenigl et al., 2021)

Voc, voriconazole; Pos, posaconazole;

### 2.3 Biological antifungals

Biological antifungals, such as monoclonal antibodies (mAbs), vaccines and antifungal peptides, are emerging as novel therapeutic options (**Table 3**). Nowadays, fungal heat shock proteins (Hsp), particularly Hsp90, are critical for the fungal stress response and survival. They are also important targets for antifungal monoclonal antibodies (mAbs).The exposed or secreted forms of Hsp90 are recognized by these mAbs, leading to the disruption of protein function, which impedes fungal growth and enhances the host immune response (Fang et al., 2025). Antifungal mAbs, such as Mycograb, are always administered alongside conventional antifungal agents like AmB. Mycograb specifically recognizes and binds to the epitope C of Hsp90 on the fungal cell wall, thereby disrupting its function, which is critical for the fungal stress response and survival, particularly under antifungal drug pressure. When used in combination with AmB, Mycograb enhances its fungicidal effects. Additionally, the presence of Mycograb supports fungal clearance by the immune system and reduces the side effects induced by AmB due to the limited drug dosage (Feng et al., 2024). The combination of Mycograb and AmB significantly decreased the mortality rate in patients with invasive candidiasis caused by *Candida* infections. This is evidenced by an increase in the survival rate from 48% with AmB monotherapy to 84% with combination therapy (Pachl et al., 2006). Relying on silico protein modeling and analysis, Ashraf S. Ibrahim’s research team identified a highly immunogenic and surface-exposed epitope in *C. auris* (hyphal-regulated protein, Hyr1p). The monoclonal antibody (mAb) was able to recognize this conserved protein across several clinical isolates of *C. auris*, preventing biofilm formation and enhancing opsonophagocytic killing of *C. auris* by macrophages. The Hyr1p-mAb significantly reduced the fungal burden and mortality rate in mice infected with lethal *C. auris* (Singh et al., 2023). Other identified antifungal mAbs that demonstrate significant fungal clearance in *C. auris*-infected mice target β-1, 2-mannotriose, hyphal wall protein 1 (Hwp1), and phosphoglycerate kinase 1 (Rosario-Colon et al., 2021, 2025). These antifungal mAbs represent a promising therapeutic option against drug-resistant strains of *C. auris*.

**Table 3:** Antifungal strategies utilizing biological antifungal agents.

Biological antifungal agents	Names and/or targets	Antifungal effects	References
Antifungal mAbs	Mycograb Target: fungal epitope C of Hsp90 on the fungal cell wall	Inhibit fungal growth and enhance host immune response Disrupt fungal cell wall and increase the vulnerability to antifungal drugs Mycograb + AmB: increase the survival rate from 48% with AmB monotherapy to 84% with combination therapy in defending invasive candidiasis	(Pachl et al., 2006; Feng et al., 2024; Fang et al., 2025)
	Target: fungal surface-exposed epitope of Hyr1p in *C. auris*	Inhibit biofilm formation and enhance opsonophagocytic killing of *C. auris* by macrophages. Rescue mice model from *C. auris* induced lethal systemic infections	(Singh et al., 2023)
	C3.1 Target: β-1,2-mannotriose	Enhance the *C. auris* phagocytosis by macrophage Inhibit biofilm formation Improve survival rate and decrease fungal burden in a complement C5-deficient A/J murine model	(Rosario-Colon et al., 2025)
	9F2 Target: phosphoglycerate kinase 1	Enhance the *C. auris* phagocytosis by macrophage Inhibit biofilm formation Improve survival rate and decrease fungal burden in a complement C5-deficient A/J murine model	(Rosario-Colon et al., 2025)
	6H1 Target: fungal Hwp1	Inhibit biofilm formation Decrease fungal burden in *C. auris* infected murine model	(Rosario-Colon et al., 2021)
Live-attenuated vaccines	PCA-2: caspofungin resistant strain of *C. albicans* CNC13: pathogenic-related genes deleted strain of *C. albicans* RML2U: pathogenic-related genes deleted strain of *C. albicans* CM1613: pathogenic-related genes deleted strain of *C. albicans*	Increases the count of polymorphonuclear cells (PMNs) and provides protection against candidiasis in murine models.	(Kaur et al., 2023)
Recombinant vaccines	NXT-2: Conserved amino acid sequences of the KEX1 regions from *Candida, Cryptococcus, Pneumocystis*, and *Aspergillus.*	Pan-fungal vaccine against *A. fumigatus, C. albicans*, and *Pneumocystis jiroyecii infections* Vaccinated mice showed significant protection when challenged with each different fungal pathogens NXT-2 elicits localized immune responses in to vaginal mucosa that reduce the fungal burden. Furthermore, it enhances the recruitment of PMNs into the vaginal lumne	(Rayens et al., 2022; Wychrij et al., 2025)
	AH-AIPO_4_: dual-antigen fusion protein vaccine for Als3 and Hyr1 proteins (Jia et al., 2024) NDV-3A:target N-terminus of Als3 (Singh et al., 2019)	AH-AIPO_4_ enhanced survival rate of *Candida* infected mice and decrease the fungal burden. It also increased Th1 and Th17 cell responses NDV-3A exhibited significant protective effects in immunosuppressed mice against *C. auris* infection	(Singh et al., 2019; Jia et al., 2024)
Liposomal vaccine platform	SNAP: targeting the short peptides of fungal cell surface proteins Fba and Met6.	Increased production of TNFα, IL-2, and IFNγ Reduce in fungal burden in the kidneys of BALB/c mice infected with *C. auris* SNAP vaccination provided protection to A/J mice against candidiasis challenges	(Huang et al., 2024)
Antifungal peptides	C14R (BP100)	Exhibited significant antifungal activity against 105 clinically isolated strains of *C. auris* with Flu resistance in Colombia. Disrupt fungal cell membrane integrity Inhibit biofilm formation	(Velez et al., 2024)
	Human cathelicidin peptide: LL-37	Disrupt fungal cell membrane integrity Enhanced fungicidal effects when used in combination with Flu or AmB.	(Rather et al., 2022)
	NFAP2	Inhibit biofilm formation and exhibit fungicidal effects in five clinical isolates of *C. auris* when in combination with echinocandins	(Kovacs et al., 2021)
	Human salivary peptide: Histatin-5	Induce ROS generation and mitochondrial dysfunction Induce necrosis in fungal cell 10 clinical isolates of Flu-resistant *C. auris* exhibited high vulnerability to histatin-5 (killing efficiency up to 90% at 7.5 μM)	(Pathirana et al., 2018; Ganeshkumar et al., 2024)
	Human neutrophil peptide-1 and human β-defensin-3	Induce both early and late apoptosis in 2 clinical isolates of *C. auris*	(Shaban et al., 2024)

mAb, monoclonal antibody; Hsp, heat shock protein; hyphal-regulated protein, Hyr1p, Hwp1, hyphal wall protein 1; KEX1, *Pneumocystis jirovecii* kexin-like protein; PMN, polymorphonuclear cell; SNAP, spontaneous nanoliposome antigen particle; ROS, reactive oxygen species; NFAP2, *Neosartorya fischeri* antifungal protein 2.

Another immunology-based therapy for the treatment of fungal infections is vaccination, which encompasses live-attenuated vaccines, pan-fungal recombinant vaccines, subunit vaccines, and conjugate vaccines (Kaur et al., 2023). The live-attenuated vaccines utilize genetically modified strains of *C. albicans* to elicit host immune protection. For example, PCA-2 is a *C. albicans* strain that exhibits resistance to caspofungin; its administration significantly increases the count of polymorphonuclear cells (PMNs) and provides protection against candidiasis in murine models. Other live-attenuated vaccine candidates include the CNC13, RML2U, and CM1613 strains of *C. albicans*, which have had pathogenic-related genes deleted (Kaur et al., 2023). NXT-2 serves as a representative example of a pan-fungal recombinant vaccine, which has been developed to provide significant immune protection against invasive candidiasis. The underlying principle of pan-fungal recombinant vaccines is to target common proteins found in various fungal pathogens. For instance, mannoproteins and β-glucans are essential for maintaining cell wall integrity and are present in numerous fungal species, making them attractive targets for the design of pan-fungal vaccines. The design of NXT-2 is based on the conserved amino acid sequences of the KEX1 (kexin-like protein) regions from *Candida, Cryptococcus*, *Pneumocystis* and *Aspergillus* (Rayens et al., 2022). In the murine model of vulvovaginal candidiasis, NXT-2 elicits localized immune responses in the vaginal mucosa that reduce the fungal burden. Furthermore, it enhances the recruitment of PMNs into the vaginal lumen (Wychrij et al., 2025). Other recombinant vaccines associated with *C. albicans* target two virulent proteins, Als3 and Hyr1, which are responsible for adhesion, biofilm formation, and evasion of the host immune response. The targeting of both adhesion and immune evasion through this dual-antigen fusion protein vaccine effectively prevents *Candida* infection and dissemination (Jia et al., 2024). The pathogenic role of Als3 has been identified in *C. auris*, analogous to its role in *C. albicans*. The N-terminus of Als3 has been developed as a vaccine (named as NDV-3A), which has demonstrated significant protective effects in immunosuppressed mice against *C. auris* infection (Singh et al., 2019). In 2024, Xin’s laboratory developed a vaccine against *Candida* by targeting the fungal cell surface proteins Fba and Met6. Short peptides derived from these two proteins were administered to a murine model using a liposomal vaccine platform known as spontaneous nanoliposome antigen particle (SNAP). The activation of SNAP was enhanced by the interaction between cobalt porphyrin phospholipid encapsulated in liposomes and three histidine residues located at the N-terminus of the synthetic short peptide immunogens. Mice immunized with SNAP-Fba+Met6 exhibited increased production of TNFα, IL-2, and IFNγ, and demonstrated a significant reduction in fungal burden in the kidneys of BALB/c mice infected with *C. auris*. Additionally, the SNAP vaccination provided protection to A/J mice against candidiasis challenges (Huang et al., 2024). The determination of targets, specifically fungal proteins and epitopes, for the design of vaccines is a fundamental step, as illustrated by the aforementioned examples. A research team from Germany has developed a methodology to identify novel CD4^+^ T cell epitopes with potential vaccine applications against *C. auris* infection. This approach utilizes the analysis of genomic databases, evolutionary information, and reverses vaccinology techniques. The team screened proteins encoded by the *C. auris* genome and identified several promising vaccine candidates. Furthermore, this methodology enables the exclusion of highly mutated or substituted epitopes that could compromise the efficacy of the vaccine (Gupta et al., 2022). It is essential to recognize that this approach is predominantly reliant on bioinformatics analysis; consequently, candidate vaccines must undergo evaluation through animal experimentation. Currently, vaccines specifically designed to target *C. auris* are less common than those developed for *C. albicans. In vivo* experiments and preclinical studies are still insufficient; thus, the evaluation of protective vaccines for *C. auris* continues to be a subject of active discussion and research.

In addition to mAbs and vaccines, small cationic peptides are also classified as biological agents that demonstrate innate antifungal activity through various mechanisms. These mechanisms include membrane disruption, inhibition of β-glucan synthase leading to cell wall disruption, induction of mitochondrial dysfunction, and the generation of reactive oxygen species. Currently, C14R, LL-37, NFAP2, and histatin-5 are well-studied antifungal peptides to against *Candida* infections (Fernandez de Ullivarri et al., 2020; Struyfs et al., 2021; Perez-Rodriguez et al., 2022; Velez et al., 2024). To address the challenge posed by multidrug-resistant isolates of *C. auris*, the research team of Firacative demonstrated that an analogue peptide of BP100, known as C14R, exhibits significant antifungal activity against 105 clinically isolated strains of *C. auris* that are resistant to fluconazole in Colombia. This activity is attributed to the peptide’s ability to disrupt cell membrane integrity and inhibit biofilm formation. The mean MIC values for these isolated strains were found to be 5.34 μg/ml (Velez et al., 2024). The human cathelicidin LL-37 peptide, similar to C14R, disrupts the fungal cell membrane, leading to rapid cell death and cytoplasmic leakage. Additionally, this peptide exhibits enhanced antifungal effects when used in combination with fluconazole or AmB (Rather et al., 2022). Furthermore, in contrast to the anti-*Candida* properties exhibited by C14R and LL-37, Dermaseptin demonstrates efficacy against four strains of *C. auris* by the stress and apoptosis. This peptide exhibits a strong antifungal activity with a MIC of 15.62 μg/ml (Wani et al., 2024). *Neosartorya fischeri* antifungal protein 2 (NFAP2) is a cysteine-rich, cationic peptide that exhibits significant anti*-Candida* activity. In conjunction with echinocandins, NFAP2 effectively inhibits biofilm formation in five clinical isolates of *C. auris* (Kovacs et al., 2021). Histatin-5, a human salivary peptide, has been identified as a potent anti-*Candida auris* agent. This peptide enters fungal cells through energy-dependent mechanisms, allowing it to bind to mitochondria. This interaction results in ATP leakage and the subsequent production of ROS, ultimately leading to cell death (Ganeshkumar et al., 2024). In the article published by Ruvini et al. in 2018, ten clinical isolates of *C. auris* were treated with histatin-5. The majority of the clinical strains, including those resistant to fluconazole, exhibited high susceptibility to histatin-5, with a killing efficiency of up to 90% at a concentration of 7.5 μM (Pathirana et al., 2018). Additionally, a study investigated the antifungal properties of human neutrophil peptide-1, human β-defensin-3, and histatin-5 against two clinical strains of *C. auris*. The results from FITC-Annexin V/PI staining and terminal deoxynucleotidyl transferase nick-end labeling (TUNEL) analysis indicated that human neutrophil peptide-1 and human β-defensin-3 induce both early and late apoptosis, whereas histatin-5 appears to trigger necrosis. However, it is important to note that this study was limited to only two clinical isolates and did not include *in vivo* experiments; thus, the full potential of these antifungal agents against *C. auris* may not be completely elucidated (Shaban et al., 2024). Although mAB, vaccines, and antifungal peptides have been extensively tested against various *Candida* species, the existing body of published studies indicates that their therapeutic efficacy against pan-resistant *C. auris* strains remains insufficient. Thus, further intensive *in vivo* and clinical studies are required to evaluate their fungicidal effects.

### 2.4 Nanotechnology-based antifungal strategies

Nanotechnology has revolutionized the delivery of antifungal agents. The limited solubility of antifungal agents, such as fluconazole, which is only slightly soluble in water, significantly restricts their clinical applications due to their poor hydrophilic characteristics. As previously mentioned, various nanoparticles have been developed to carry or encapsulate antifungal drugs, thereby enhancing their bioavailability and antifungal efficacy. For instance, orally administered AmB nanoformulations from Martina Biopharma Inc. have undergone a Phase I clinical trial, demonstrating no severe side effects at oral doses of 200, 400, and 800 mg over a two-week period. These oral AmB nanoformulations have been evaluated for their ability to eliminate *Cryptococcus* from cerebrospinal fluid (CSF) in HIV patients and to treat vulvovaginal candidiasis (VVC) at dosages of 200 and 400 mg. However, the antifungal efficacy of these nanoformulations against *C. auris* and their final approval for clinical use may still necessitate further intensive research (Fairuz et al., 2022). Lipid-based nanoparticles, such as AmBisome^®^, are extensively utilized for intravenous administration in the treatment of infections caused by *Cryptococcus*, *Aspergillus*, and *Candida* species. According to the recommendations from the CDC, patients should receive a dosage of 5 mg/kg intravenously once daily for the treatment of *C. auris* infection, particularly in cases where there is a lack of response to echinocandin therapy or in instances of persistent fungemia lasting more than five days. Additionally, recent advances include the use of lipid nanoparticles and metal-organic frameworks (MOFs) to enhance the bioavailability and efficacy of antifungal drugs were summarized in **Table 4**. Targeting *C. auris*, nanoparticle delivery systems include lipid-based, and metal-based; both of these systems exhibited enhanced antifungal efficacy, particularly in inhibiting biofilm formation (Sutar et al., 2022; Fayed, 2024). Interestingly, one study demonstrated that silver nanoparticles exhibited fungistatic activity even without the incorporation of antifungal drugs (AlJindan and AlEraky, 2022). Moreover, zinc oxide nanoparticles serving as carriers for caspofungin demonstrated enhanced antifungal efficacy against caspofungin-resistant strains of *C. auris* (Fayed et al., 2021). Additionally, chitosan–PLGA (polylactide-co-glycolide) loaded with fluconazole exhibited improved drug encapsulation and prolonged release of azoles. The formulation of fluconazole loaded in chitosan-PLGA demonstrates a 64-fold increase in antifungal efficacy compared to fluconazole alone. Moreover, this fluconazole-chitosan-PLGA system showed significantly enhanced antifungal efficacy against fluconazole-resistant *C. auris* and *C. albicans* in both *in vitro* and *in vivo* models by inhibiting biofilm formation. Furthermore, the nephrotoxicity and hepatotoxicity associated with this nanoparticle-based delivery system are negligible in these nano-drug delivery systems (Kolge et al., 2023). Another critical nano-system that combats fungal infections is the lipid-based system, commonly referred to as liposomes (Marena et al., 2024). This system demonstrates significant advantages in the sustained release of drugs and enhanced bioavailability of antifungal agents. Consequently, lipid-based nano-systems have received approval from the FDA for the treatment of invasive candidiasis and aspergillosis, specifically the formulations AmBisome^®^, and Abelcet^®^, which contain amphotericin B (AmB) (Adedoyin et al., 2000; Stone et al., 2016; Bruggemann et al., 2022). Furthermore, the aforementioned marketed liposomal AmB formulations demonstrate properties that mitigate the nephrotoxicity associated with AmB in patients (Olson et al., 2008). Although these FDA-approved lipid-based nanosystems demonstrate significant antifungal efficacy against *Candida* and *Aspergillus* species, evidence regarding their fungicidal effects on drug-resistant strains of *C. auris* remains lacking.

**Table 4:** Nanotechnology-based antifungal strategies.

Nano systems	Outcomes	Clinical Implications in targeting antifungal resistant *C. auris* strains	References
Silver Nanoparticles	Disrupt fungal cell membranes via ROS generation and inhibit biofilm formation	Only preclinical studies exhibited efficacy against the growth and biofilm formation in multi-drug resistance strains of *C. auris* No registered clinical trials yet	(Vazquez-Munoz et al., 2020; Fayed, 2024)
Zinc Oxide Nanoparticles	Act as a carrier for delivering caspofungin and cause the damage of cell walls	*In vitro* and *ex vivo* tested only	(Fayed et al., 2021)
Chitosan-Based Nanoparticles	Serve as a carrier for the delivery of fluconazole, which inhibits the growth of multidrug-resistant (MDR) strains of *C. auris*.	*In vitro* and *in vivo* tested only	(Kolge et al., 2023)
Polymeric Nanoparticles	Serve as a carrier for loading fluconazole and	No registered clinical trials yet	(Leon-Buitimea et al., 2021; Kolge et al., 2023)
Marketed Lipid-Based Nano-systems	AmBisome^®^ is the lipid-carrier AmB and has been used to treat systemic fungal infections Abelcet ^®^ is the Liposomal AmB and has been proved to treat the invasive candidiasis	No clinical trials to *C. auris*-specific resistance	(Adedoyin et al., 2000; Stone et al., 2016; Bruggemann et al., 2022)

### 2.5 Combination therapies

Combination therapies utilize various FDA-approved antifungal drugs to treat fungal infections, demonstrating significant efficacy in addressing resistant fungal strains (**Table 5**). For resistant *C. auris*, combination therapies have shown promise in overcoming antifungal resistance, and several clinical studies have reported positive outcomes from the combination of azoles and echinocandins (Griffith and Danziger, 2020). One study demonstrated the effectiveness of combining new drugs (diphenyl diselenide and nikkomycin Z) with currently used antifungals against *C. auris*. These combination therapies significantly inhibited the growth of *C. auris.* However, the monotherapy of these two new drugs exhibited less antifungal efficacy (Poester et al., 2022). A research team from Brown University discovered that benzodiazepines can improve the effectiveness of fluconazole and reestablish the sensitivity of azole-resistant *C. albicans* isolates. Notably, the exclusive application of this small molecule did not exhibit any toxic effects on fungal cells. Although this study focuses exclusively on *C. albicans* and does not employ animal infection models, it proposes a potential method for the synergistic use of small molecules to enhance the antifungal efficacy of traditional antifungal agents (Alabi et al., 2023). Furthermore, in addition to the combinations of two or more antifungal agents, a research team from the United States demonstrated that the synergistic use of AmB and an antiemetic drug exhibits significant anti-*C. auris* activities by enhancing the fungicidal activity of AmB in a very short time. They screened over 2,600 FDA-approved drugs and clinical compounds to identify rolapitant as a promoting agent that enhances antifungal effects by inducing oxidative stress, interfering with ATP production, and compromising the mitochondrial functions of *C. auris* (Salama et al., 2024). The same team identified that the combination of HIV protease inhibitors (lopinavir and ritonavir) with azoles (e.g., fluconazole and itraconazole) significantly reduces the burden of resistant strains of *C. auris* in mice (Salama et al., 2023).

**Table 5:** Combination therapies for treating *C. auris* infection.

Combination Therapies		Outcomes	Clinical Implications in targeting antifungal resistant *C. auris* strains	References
5-flucytosine (5-FC) or manogepix	anidulafungi, AmB or voriconazole	Anidulafungin + manogepix or 5-FC exhibit great antifungal efficacy to resistant *C. auris* strains	*In vitro* tested only A total of 25 clinical strains of *C. auris* have been isolated, categorized into clades I, III, and IV.	(John et al., 2023)
AmB	Rolapitant	Rolapitant restored the fungicidal effects of AmB within 4 h. Rolapitant + AmB significantly reduce the fungal burden in *C. auris* infected mice model.	In vitro and in vivo tested A total of 29 clinical strains of C. auris with AmB resistance have been tested.	(Salama et al., 2024)
Lopinavir and/or ritonavir	Fluconazole or itraconazole	Lopinavir or ritonavir exhibited synergistic interactions with azole to against 91% of *C. auris* strains Ritonavir interfere the fungal efflux pump Both of lopinavir and ritonavir boost the antifungal effects of azoles in mice model	In vitro and in vivo tested A total of 34 azole resistant strains of C. auris were tested	(Salama et al., 2023)
18 Human intravenous immunoglobulins (IVIG, IgG) were obtained from random healthy donors	AmB	IVIG + AmB extend the lifespan of multidrug-resistant *C. auris* and *C. albicans* infected mice Candida Peptide- and glycan-related IgGs of IVIG displayed the highest protection effect	*In vivo* tested: *Candida* infected mice model *Two C. auris* strains: AR-CDC0387, AR-CDC0386, and one *C. albicans* strain: ATCC MYA-2876 were tested	(Xin et al., 2023)
Chlorhexidine acetate	Fluconazole	The findings demonstrated that the 80% MIC for fluconazole alone against *C. auris* ranged from 2 to 32 mg/L, while those for chlorhexidine acetate ranged from 2 to 8 mg/L. Chlorhexidine acetate + fluconazole significantly inhibit the *C. auris* biofilm formation	*In vitro* tested only *20 clinical strains of fluconazole-* resistant and fluconazole-susceptible *C. auris* were tested	(Hao et al., 2022)
Anidulafungin, AmB, voriconazole or Manogepix	Manogepix or 5-FC	The combination of anidulafungin with manogepix or 5-FC displayed the highest potential against the tested *C. auris* isolates.	*In vitro* tested only *A total of 25 clinical strains of C. auris* with fluconazole resistance were isolated from 6 patients	(John et al., 2023)
Echinocandin (caspofungin or micafungin)	Voriconazole or fluconazole	Synergistic utilization exhibit	*In vitro* tested only A total of 10 clinical and multidrug-resistant (fluconazole and micafungin-resistant isolates) *C. auris* strains from hospitals	(Fakhim et al., 2017)
Anidulafungin	Voriconazole or isavuconazole	Isavuconazole combined with anidulafungin demonstrated greater antifungal efficacy than voriconazole combined with anidulafungin against drug-resistant strains of *C. auris*.	*In vitro* tested only	(Pfaller et al., 2021)
Anidulafungin	Caspofungin	One out of two patients survived the infection caused by *C. auris.*	Case report	(Mohsin et al., 2017)
5-FC	AmB, voriconazole or micafungin	Improved fungal clearance	*In vitro* tested only	(Bidaud et al., 2019)
5-FC	AmB, azoles or echinocandins	For AmB-resistant strains: AMB + 5-FC exhibit 100% inhibition For echinocandin-resistant strains: anidulafungin/caspofungin/micafungin + 5FC exhibit 100% inhibition For voriconazole-resistant isolates voriconazole exhibit 100% inhibition	*In vitro* tested only *1,000 isolated C. auris* with fluconazole resistance	(O’Brien et al., 2020)
Sulfamethoxazole	Azole (fluconazole, itraconazole, or voriconazole)	*In vivo* testing in *Caenorhabditis elegans (C. elegans)* demonstrated a 70% increase in survival with the combination of sulfamethoxazole and voriconazole.	*In vitro* and *in vivo* (*C. elegans* infection model) experiments *10 isolated clinical strains of C. auris*	(Eldesouky et al., 2018)
Miltifosine	AmB or fluconazole	A combination of miltifosine and AmB exhibits fungicidal activity against 25% of fluconazole-resistant *C. auris* strains; however, a combination of miltifosine and fluconazole does not demonstrate improved fungicidal activity.	*In vitro* tested only *12 isolated C. auris* with fluconazole resistance	(Wu et al., 2020)
AmB	Anidulafungin	One patient who suffered from an invasive C. auris infection survived.	Case report	(Reque et al., 2022)
Micafungin	Caspofungin	The 30-day mortality rate associated with bloodstream infections caused by C. auris exceeds 30%.	Multicenter study	(Simon et al., 2023)
Fluconazole	AmB	Two out of three patients survived the bloodstream infection caused by *C. auris.*	Case report	(Lee et al., 2011)

### 2.6 Photodynamic-based therapies

Photodynamic therapy (PDT) has been widely used in cancer treatment since the 1970sand demonstrated significant efficacy in the treatment of superficial fungal infections, particularly against *Candida* infections. Research conducted by Hamblin et al. has focused on the application of PDT for the treatment of cutaneous infections caused by *C. albicans*. The study identified that the combination of methylene blue and red light (630 nm, 90 seconds per session, 4.5 J/cm²) is highly effective in reducing the fungal burden on the skin in murine models by 90%. Furthermore, this approach exhibited 100% efficacy in preventing *C. albicans* infections, positioning it as a rapid, non-invasive treatment strategy. Methylene blue serves as a critical photosensitizer in this synergistic approach to combat cutaneous fungal infections (Dai et al., 2011). Recently, due to the increasing prevalence of fungal infections and the rising incidence of antifungal drug-resistant cases, several research groups have sought to utilize PDT to treat *C. auris* infections (Bapat and Nobile, 2021). Bapat et al. investigated the antifungal effects of blue light alone, red light in conjunction with a photosensitizer, and green light combined with a photosensitizer. The results indicated that blue light alone exhibited the most significant inhibitory effect, reducing biofilm formation by 77% and disrupting mature biofilms by 57% after a 24-hour exposure, in comparison to the other two light treatments. Furthermore, the combination of blue light with photosensitizers, such as toluidine blue O, enhanced biofilm inhibition by an additional 7-22%. In the cellular and molecular level, PDT works by damaging various molecules within fungal cells through the stimulation of ROS generation. Unlike conventional antifungal agents, which target specific molecules, PDT minimizes the risk of inducing drug resistance. For instance, one study introduced the cage-modified hypocrellin as an innovative antifungal compound that demonstrates significant effectiveness against multidrug-resistant *Candida* species. This efficacy is underpinned by a well-defined mechanism of action, offering a promising therapeutic strategy for tackling resistant fungal infections (Liu et al., 2022). Although PDT has the potential to combat drug-resistant *C. auris* strains, several limitations must be acknowledged. These include the limited tissue penetration depth of activating light wavelengths, reduced efficacy against biofilm-embedded fungi due to impaired diffusion of photosensitizers, oxygen dependency in hypoxic infection sites, potential photo-toxicity to host tissues, and insufficient residual activity against fungal regrowth following treatment. These factors necessitate repeated applications, complicating clinical implementation.

## 3 Future directions

Despite significant progress in the development of alternative antifungal strategies for *Candida auris*, several critical gaps and challenges persist. The intricate nature of antifungal resistance mechanisms, combined with the organism’s capacity to thrive in various clinical settings, underscores the need for a comprehensive research agenda. Addressing the disparity between promising laboratory results and effective clinical applications will necessitate innovation in both scientific and translational domains. Several avenues for future research can be identified: 1. Application of artificial intelligence (AI) and machine learning (ML) methods for the selection of novel antifungal agents; 2. Addressing challenges in clinical translation; and 3. Enhancement of resistance monitoring and diagnostics.

AI has undergone rapid development over the past decade, significantly influencing various fields worldwide. ML, a subset of AI, employs statistical models and algorithms to analyze data. The application of AI and ML methodologies is anticipated to accelerate the discovery of novel antifungal targets and the optimization of antifungal drugs (Fu et al., 2021; Thorn and Xu, 2025). However, the transition of alternative therapeutic agents from laboratory research to clinical application is hindered by challenges such as *in vivo* toxicity, pharmacokinetics, formulation stability, and the regulatory approval processes for novel compounds and delivery systems. There is an urgent need for more comprehensive preclinical evaluations and the development of optimized formulation strategies. Additionally, proactive engagement with regulatory agencies is essential to clarify the approval pathways for emerging classes of antifungal agents. The development and implementation of rapid, real-time diagnostic tools for resistance profiling in clinical isolates are critical. The establishment of enhanced surveillance models will facilitate the early detection of resistance trends and support tailored therapeutic strategies (Cornely et al., 2025).

In conclusion, addressing the drug resistance of *C. auris* necessitates sustained innovative research into alternative therapeutic options, as well as decisive measures to overcome translational, diagnostic, and regulatory challenges. By synthesizing new scientific knowledge with cutting-edge technologies and collaborative frameworks, the field is strategically positioned to achieve significant advancements in combating this emerging threat.

## 4 Conclusion

Lethal infections caused by fungal pathogens pose a significant threat to human health, and the issues of antifungal resistance and the limitations of conventional antifungal therapies should not be overlooked. Among the most dangerous fungi, *Candida auris* has been associated with rising mortality and incidence rates worldwide. Many clinical isolates of *C. auris* exhibit natural resistance to antifungal drugs, including azoles, polyenes, and echinocandins. Consequently, numerous studies are focused on developing alternative antifungal strategies, which include the creation of novel antifungal drugs, the identification of natural antifungal agents, the design of nanotechnology-based drug delivery systems, and the exploration of combination therapies, among others. In this review, we have highlighted the antifungal mechanisms and advancements associated with alternative therapies. Natural antifungal compounds demonstrate fungistatic effects against various multi-drug resistant clinical strains of *C. auris*, suggesting that not only chemically synthesized compounds can be classified as antifungal agents. Novel synthetic antifungal compounds, such as isavuconazole, possess distinct structures that interact with lanosterol 14α-demethylase and inhibit the expression of efflux pumps in *C. auris.* Fosmanogepix treats candidemia by inhibiting the Gwt1 enzyme, thereby disrupting the transport of mannoproteins within the fungal cell wall and membrane. These innovative synthetic antifungal agents effectively circumvent conventional drug targets, thereby overcoming drug resistance. The antifungal mechanisms of biological agents, vaccines, peptides, and monoclonal antibodies (mAbs) rely on the host immune system to eliminate *C. auris* strains. Combination therapeutic strategies are frequently employed in antifungal treatments, as synergistic administration and dosage restriction of antifungal agents can reduce toxicity, enhance antifungal efficacy, and particularly mitigate the development of acquired drug resistance. Nanotechnology-based delivery systems aim to improve the bioavailability of antifungal drugs, significantly reducing the required dosage and the emergence of drug resistance. Finally, while photodynamic therapies utilize physical methods to directly eradicate *C. auris* and prevent drug resistance, the antifungal efficacy of these approaches for invasive candidemia requires further investigation.

While these strategies have shown promising fungistatic and fungicidal effects against clinically isolated multidrug-resistant *C. auris* strains, the potential antifungal effects must be rigorously tested in animal models and human subjects. Additionally, the pharmacodynamics and pharmacokinetics of these antifungal agents should be evaluated, and any potential side effects must be identified. Consequently, with the rising incidence of infections, the increased consumption of antifungal medications, and the growing issue of antifungal drug resistance, the development of alternative antifungal therapies remains an emerging area of research.
